# AI-generated corpus learning and EFL learners’ learning of grammatical structures, lexical bundles, and willingness to write

**DOI:** 10.1371/journal.pone.0321544

**Published:** 2025-07-11

**Authors:** Chunlin Lu

**Affiliations:** 1 School of foreign languages, Baotou Teachers’ College, Baotou, China; National University Philippines, PHILIPPINES

## Abstract

This research study examined the manner in which English as a Foreign Language (EFL) learners’ willingness towards writing, grammatical construction, and lexical bundle acquisition were affected by language of AI-generated corpora. Eighty EFL students from China’s Baotou Teachers’ College participated in a repeated-measures quasi-experimental study and were split into controlled and trial groups. During the span of the 14-sessions, the experimental cohort underwent education that utilized AI-driven corpora, whereas the controlled cohort was instructed through traditional textbooks. Data were collected using pre- and post-treatment assessments, including tests on lexical bundles and grammar, as well as a willingness to write scale. The findings revealed that the trial group’s performance was substantially improved than the controlled cohort regarding all outcomes. Specifically, trial group demonstrated higher mean scores for lexical bundles, grammar, and willingness to communicate. Statistical analysis, including mixed ANOVA, confirmed the significant effects of time and group membership on language proficiency and willingness to communicate. These findings suggest that the intervention positively influenced learners’ language skills and attitudes toward writing. The interaction effects of time and group membership further highlighted the nuanced relationship between instructional interventions and learner outcomes. The study underscores the importance of integrating AI-driven language corpora into language teaching to enhance vocabulary, grammar, and communication skills. Pedagogical implications include the need for dynamic and engaging learning environments, while curriculum developers should consider incorporating data-driven learning approaches. Further research might identify optimal designs for learning and investigate its long-term impacts associated with such interventions. Policies should support equitable access to technology-enhanced language learning resources, promoting more effective language education practices overall.

## 1. Introduction

A multifaceted landscape filled with possibilities and complications is revealed by scientific research on integrating artificial intelligence (AI) tools into educational contexts, notably large language models (LLMs) such as ChatGPT. ChatGPT may demonstrate motivation, involvement, and acquisition of language. Moreover, incorporating AI technology into education can improve learning for students [1]. Debates over academic integrity and ethical issues are exceeding because AI becomes increasingly prevalent in academia. Grassini [2] advocates for openness in the way students use large language models (LLMs) including ChatGPT and suggests that academic credibility regulations should be upgraded to particularly confront this use. Corresponding to this, Moya et al. [3] express concerns regarding educational impartiality and honesty call for the creation of precise rules and regulations to guarantee the ethical and accountable application of AI techniques in learning environments. In tandem, these insights highlight the necessity of a well-rounded strategy that minimizes any integrity and ethical problems whilst optimizing the advantages of AI in instruction.

The field of language instruction and acquisition has changed recently mainly the consequence of the combination of corpus the study of language and generative AI (GenAI) technological advances. Corpora linguistics, with its emphasis on data-driven language analysis, has long been fundamental to understanding language patterns and usage across diverse contexts. Concurrently, GenAI applications like OpenAI’s ChatGPT have revolutionized human-computer interaction, offering real-time conversation, formative feedback, and instant text generation tailored to specific registers and genres [4,5]. This convergence prompts a critical examination of the potential synergies between corpora and GenAI in data-driven language learning (DDL). Corpora provide invaluable insights into language usage, offering authenticity, replicability, and multimodality. They inform pedagogical practices aimed at enhancing language proficiency and literacy. On the other hand, GenAI technologies offer unique advantages in user experience, differentiation, and data size, ushering in new possibilities for language education [6]. However, challenges and limitations accompany each approach. Corpus-based DDL often requires technical expertise and suffers from complex interfaces and limited tracking capabilities. Conversely, GenAI outputs may lack authenticity and suffer from hallucinations or inaccuracies, posing challenges for learners and educators alike [7]. Therefore, there is a need for a comprehensive framework that integrates corpora and GenAI in DDL. The challenges with language are exacerbated in educational environments by the requirement to become proficient in subjects unique. Comprehending the course contents requires a solid command of specialized language and concepts of technology. The phrases, style and contents tend to be confusing to learners who are not native and not used to English language [8]. In addition to inadequate levels of familiarity to professional English as well as deficiency of useful coping mechanisms for new words, students of EFL usually have trouble understanding what they are reading across broad learning environments [9]. Additionally, since globalization keeps influencing instructional institutions, learners being able to communicate using English growing its importance for their ability to take advantage of internationally recognized research, engage with counterparts from around globe, and maintain recent about developments to their chosen fields [10].

Technical obstacles should be addressed, data security and reliability should be guaranteed, and innovative thinking and interactive learning should be encouraged. Instructors and investigators can offer individualized, successful, and captivating language acquisition encounters for students of every stage of proficiency by combining the advantages of both techniques [11]. The potential advantages of AI incorporation in linguistic instruction for acquiring vocabulary, competence in languages improvement, and engagement among learners have been addressed in a number of earlier research investigations [12]. Study has shown that AI-driven corpora are useful for improving learners’ understanding of specialized nomenclature and encouraging retention of vocabulary [13,14]. Moreover, research by Seo et al. [15] has indicated that AI-driven platforms can positively influence learners’ academic emotions, fostering a more positive learning environment.

Large language models (LLMs) such as ChatGPT, especially, have a capacity to improve motivation for learners, enthusiasm, and learning of languages, corresponding to the material currently available regarding the use of AI technologies in instructional conditions. Nevertheless, intellectual, and moral challenges still exist, highlighting the necessity of revised rules and regulations to guarantee the proper and fair application of AI techniques. Language teaching and acquisition have changed recently due to the confluence of corpora of language and generative AI (GenAI) tools. While corpora provide valuable insights into language usage and inform pedagogical practices, GenAI applications offer unique advantages in user experience and data size. Yet, every technique has drawbacks like credibility problems as well as technical bottlenecks, highlighting the significance towards a robust structure that combines corpora and GenAI via data-driven learning of languages (DDL). The benefits powered by artificial intelligence learning language techniques have been examined in previous studies, with particular attention paid to topics including growing vocabulary, improving conversational competency, and encouraging student involvement. However, it appears an insufficient of understanding for the exact effect of AI-powered corpora upon students of EFL in general.

The current research studies have not adequately addressed the three characteristics likewise learners’ acquisition of grammatical structures, lexical bundles, and writing willingness are not adequately addressed by the current research studies. The restrictions may arise from their inability to generate wisdom in their collection of questions to address the main issues facing the students. While some research papers have evaluated the AI-generated corpus in isolation to a few aspects, others have not included the “willingness to write” feature in their analyses. This research study filled the gap in order to address the uniqueness. This study is essential for investigating how artificial intelligence-driven corpora greatly affect students learning English as a foreign language’s ability to understand and retain lexical bundles. Furthermore, the main factor that is compelling this research study is the substantial influence that artificial intelligence-driven corpora have on EFL students’ acquisition of grammatical structures.

By going through the existing research studies along with examining how AI-driven corpora affect EFL learners’ acquisition of vocabulary, lexical bundles, and writing motivation, the current study fills this research gap. By examining the potential benefits of incorporating AI-driven corpora into EFL instruction, the study contributes to our understanding of effective language learning strategies. Specifically, the findings are expected to shed light on how AI-driven technologies can enhance learners’ ability to identify and utilize relevant lexical bundles, facilitating more effective communication within their professional domain. This endeavour also seeks to determine how AI-driven corpora affect the willingness of students to write, offering insightful information to designers of curriculum and instructors of language. In overall, this investigation has impact on the development and application of technologically enriched language immersion settings, fostering individualized, successful, and captivating linguistic educational opportunities regarding EFL students. The current study examines how AI-powered corpora affect EFL students to acquire vocabulary, lexical bundles, and writing motivation. It is anticipated that the results of the present research will provide insight on the possible advantages of using corpora of AI-driven during EFL learning.

## 2. Review of related literature

The recent introduction of OpenAI’s ChatGPT in the year 2022 has been followed by a subsequent surge in the utilization of generative artificial intelligence (GenAI), leading to a notable and undeniable shift in the perceptions held by the general populace with regards to the potentialities inherent in human interaction with extensive language datasets. This shift has brought to light a new realm of exploration and understanding that corpus linguists have been diligently working on for several decades, as demonstrated by their persistent efforts dating back to the early 2000s [16]. The effects of this shift are especially noticeable for those who work diligently within the arena about corpus-oriented data-driven learning (DDL), which is currently steadily gaining prominence and curiosity in recent years. It is crucial to note that employing of datasets to improve linguistic learning and teaching possesses a rich and lengthy record spanning more than 20 years, and its development has significantly influenced pedagogical approaches and techniques. By stimulating an approach of research, examination, and exploration with real objects, DDL helps students analyse language through corpora data and fosters independence in acquiring a specific language. Furthermore, DDL aims to cultivate continuous and proactive researchers that is considered an essential quality for professionals amid a globe that is continuously changing [17]. DDL practices are believed to improve the language acquisition process by giving learners the opportunity to see how different language patterns are used frequently and prominently. This exposure can happen through either independent engagement with learning methods that involve active participation or in social settings like classrooms, where learners work with their peers and teachers on activities that use corpus data, as discussed by O’Keeffe [18]. Numerous research studies have shown the advantages of incorporating DDL into language learning for a wide range of linguistic goals in controlled environments. Objections and worries regarding the efficacy in corpora technologies and the accuracy of corpora data in addition to positive outcomes have also serious challenges [19].

The utilization of DDL techniques in language learning has been shown to have a positive impact on learners by exposing them to various language patterns that are commonly used and stand out. This exposure can be achieved through independent learning methods that encourage active participation or in social environments like classrooms, where learners collaborate with their peers and instructors on activities that involve corpus data, as indicated by O’Keeffe [18]. Extensive empirical studies have demonstrated the advantages of integrating DDL into language learning for a diverse set of linguistic goals in controlled settings. These studies have generally found a favorable perception of DDL, as highlighted by Boulton & Vyatkina [17]. However, while these positive outcomes are prevalent, there are also criticisms and reservations regarding the effectiveness of corpus tools and the accuracy of corpus data, as expounded upon in detail by Ma et al. [19]. In real time debate, instantaneously instructive and constructive criticism, authentic language insights for vocabulary through details, instantaneous writing of particular files and categories, encyclopedias entries and illustrations, along with translation by machine are some of the envisaged features regarding GenAI during the acquisition of languages [18]. Despite these promising benefits, their practical application in classroom settings has been limited by the time it takes to deploy GenAI applications like ChatGPT, necessitating further empirical investigation [20].

However, existing scholarly literature has extensively documented the educational benefits of utilizing corpora to enhance understanding across different genres and registers, bolster vocabulary acquisition, improve grammar proficiency, and facilitate error correction. This evidence is supported by researchers such as Ma & Mei [19], Gablasova et al. [21] and Poole [22]. The participants during experimental group writing abilities significantly improved, as evidenced by rising averages as well as difference that is statistically significant. The willingness of learners to write are greatly enhanced when AI-powered instruments are incorporated into professional English writing instruction. The incorporation of artificial intelligence in English writing courses may be a useful way to overcome difficulties within the educational environment and improve student learning outcomes. This study suggests a broader use of AI application during classroom to help pupils improve their writing abilities [23]. In the age of GenAI, it makes sense no reason whatsoever to stop this behaviour. Examining the many advantages that corpora technologies still have above GenAI programs which resemble chatbots that is vital. Analysing data represents a few main advantages. Although researchers hold thorough comprehension of the language and realm through which the corpus information comes, corpora are proving to serve as an essential investigation and pedagogical instrument. The immense models of language which currently underpin programs including ChatGPT are unable to provide this degree of understanding. Researchers can obtain the whole transcripts from certain huge generalised corpora, similar to BAWE and BNC2014, because they belong to recognised facts. In this regard, Zaki [24] offers a pedagogical demonstration of the method of teaching. Additionally, ‘citation’ features provided by tools such as CorpusMate allow consumers to figure out the corpora wherein the material was taken, the topic of the corresponding writing, and a connection to the database. The capacity to create do-it-yourself corpora, as demonstrated by Charles [25], presents a significant advantage in this context. Learners can possess full control over the corpus utilized for subsequent investigations.

According to Ngo et al. [26], automated writing evaluation (AWE) proved less successful at enhancing learners improve their writing grammar however more successful at enhancing the utilization of vocabulary. Grammarly is likely to be an invaluable instrument. In comparison to non-AWE intervention, the medium-to-long time frame for AWE application exhibits a stronger effect, while short-term use results in a lesser influence on writing results. Moreover, learners having intermediate English competence, undergraduates, and learners in a situation involving EFL all benefit from AWE. When all factors considered, AWE is a useful tool that should be regularly used in courses for writing. The integration of artificial intelligence (AI) tools, particularly large language models (LLMs) like ChatGPT, into educational settings presents both opportunities and challenges. One key consideration is the authenticity of the language generated by these tools. Unlike corpus data produced by humans, GenAI output may lack contextual or register-appropriate language, requiring subsequent tweaking to minimize discrepancies [27]. It becomes problematic, especially for those studying second languages whom depend on real linguistic information for retention.

Replicability is still another important factor. Although GenAI programs utilise intricate statistical processes to produce text, users are unable to duplicate these processes or get the same outcomes for a similar search [28]. Contrary, corpora have the benefit of reliably reproducing results, which offers concrete proof of language acquisition [29]. The dependability of corpora as teaching tools is increased by this replication ability. The beneficial effects of this kind of integration are regularly emphasized by studies by Muñoz et al. [1], which show increases in student involvement, task inspiration, and argumentation abilities. Such findings highlight how chatbots might improve interactive learning environments. At the same time, Hong [30] promotes conversations about the moral and responsible use of chatbots in education by highlighting the substantial potential that ChatGPT offers for teaching and evaluating second as well as foreign languages. Mohammadkarimi [31] agrees that emphasizing the value of extensive education and ethical issues for educators while utilizing AI technologies.

Hutson et al. [32] acknowledge the benefits of AI models such as ChatGPT-3 in poetry analysis, but caution against ignoring the inherent human feelings and experiences that are essential to creativity. Ray and Das [33] emphasize the need for a multifaceted strategy to address both ethical and realistic obstacles that arise from the inclusion of AI in education, share this viewpoint. Additionally, Jeon and Lee [34] outline the many functions of ChatGPT in learning environments, emphasising the value of instructors’ pedagogical knowledge in utilising AI technologies successfully. This claim is further supported and proposed the educational initiatives and tailored support systems designed to address the challenges of implementing AI in educational institutions [35,36]. The function using ChatGPT, the OpenAI consortium conversational artificial intelligence (AI), in acquiring languages crucial for upcoming technologies. The first section describes ChatGPT’s structure emphasizes its usefulness tool for both teachers and learners of languages. These qualities include access, personalization, fully immersive instruction, and quick feedback. Consequently, it works best when used in conjunction with conventional teaching to create a mutually advantageous connection that enables students to learn languages. Certain constraints and related difficulties with using ChatGPT during language acquisition environments are needed to be considered [37].

In their study, Zamanpour & Etemadzadeh [38] discovered that the Artificial Intelligence Driven Corpus (AIDC) has the ability to enhance learners’ involvement, enthusiastic feelings, and utilization of lexical bundles along with idioms. Additionally, students’ negative feelings were lessened by the intervention. These results imply that, especially in specialized domains like financial technology, including AIDC towards language training might resulted in greater lexical learning, higher learners’ dedication, and enhanced mental wellness in general. In the field of language acquisition, Klimova et al. [39] stress the value of chatbots’ brevity, augmented reality, and instant feedback, highlighting how these features might improve student performance. ChatGPT has the capacity to assist students of English improve their command of the language by serving as a useful grammar-checking instrument [40]. Nonetheless, Mohamed [41] discusses the importance of addressing ethical issues related with the usage employing AI methods throughout teaching. Such debates highlight the necessity of current academic honesty guidelines and continuous discussion concerning the application of AI responsibly.

However, the study of Bui and Barrot [42] observed that ChatGPT’s assessment were inconsistent after two sessions of grading (weak correlation coefficients between classes scores) and didn’t correspond to those of a competent human assessor (moderately weak associations). ChatGPT’s score system, data collected for training, updates to models, and intrinsic randomisation were the main causes of their results. Considering the appropriate precautions to guarantee the authenticity of written form, ChatGPT is capable of being used as an instrument throughout the process of composing. In order to find discrepancies, L2 writing experts can also examine the grammar and syntax of writings produced via ChatGPT while contrasting these against works composed by human authors. This approach, when evaluating writing, learners might be educated to identify an AI-generated its entirety. Through consistently giving learners input on their work without relying on preconceived notions, artificial intelligence (AI) systems like ChatGPT offer the ability to promote transparent impartial writing screening. Along with offering promptly individualized feedback which improves the learning of students [43].

### 2.1. Studies on willingness to write

Composition is a complex process that includes coming up with opinions, exploring one’s own thinking, and creating significance [44]. The first language (L1) along with the second language (L2), such an approach poses significant difficulties because authors have to negotiate a number of components essential to clear discourse [44]. Producing writings are generally regarded among the hardest abilities for language students to achieve proficiency [45,46]. Yavuz and Genc [47], in an EFL setting, found that writing compositions are perceived as highly daunting by most learners, regardless of their proficiency level, often resulting in a mere attempt to pass examinations. Moreover, teachers in this study noted students’ lack of enthusiasm for writing during classes and their reluctance to engage in even basic writing tasks [47]. Brown [48] describes writing as a struggle, a sentiment echoed by teachers grappling with the same challenges. In the contemporary technological landscape, educators face difficulties in motivating reluctant writers to embrace creative approaches to writing [49]. The challenge in acquiring abilities to write is highlighted by Klimova [50], who cites societal variables like poor judgement towards the desired language including perceived stagnation, among other cultural variation to comprehend academic vocabulary patterns spanning varied states. Feedback emerges as a pivotal factor in enhancing students’ motivation in writing, as evidenced by a study on international students in Thailand [51]. Similarly, a study on Saudi student writers identified challenges such as idea generation, accuracy, and meeting teacher expectations as significant stressors [52]. Leki [53] highlights the myriad challenges faced by writing instructors, from practical issues like class size and time constraints to ideological hurdles such as institutional support and resistance to prescribed teaching methods.

Willingness to communicate (WTC) which is described simply a desire to start interaction once offered the opportunity, was first investigated within the realm that first- language communications and later broadened to second-language acquisition [54]. One of the key elements that propels students achieving being proficient in L2 involve (WTC) [55]. Even though crafting sentences in English remains a crucial ability across ESL or EFL settings, students frequently show little excitement or inspiration for it. This disparity is made worse by a number of reasons, especially apathy, scepticism, and detachment [54].

A relatively recent addition to the discipline of practical linguistics, the acronym “lexical bundle” implies a group comprising a minimum two words that frequently occur simultaneously during a fixed-string but have no colloquial [56]. Expressions like “in the current study,” “as a result,” and “on the one hand” have been frequently used samples involving lexical bundles. Despite the fact offering lexical combinations are somewhat common in languages [57], 10 also notes that learning and applying lexical bundles effectively seldom occurs as one may anticipate. Numerous academics have come to the conclusion that whereas researchers use a wide variety of lexical bundles pursuing their written work to support positions and persuade audience members, learners of similar subjects do not use similar methods [57,58].

Numerous facets of integrating AI into language learning have been examined in earlier studies, which have highlighted the potential advantages for learning new vocabulary, competency in languages improvement, and engagement among learners [59,60]. Researchers [11, 12] have observed that how well AI-driven corpora support retained vocabulary as well as boost learners’ understanding of specialised terms. Furthermore, studies by Seo et al. [15] have shown that AI-powered platforms can have a favourable impact on students’ academic feelings and create a healthier atmosphere for learning. Although literature currently in production offers insightful information about the advantages of AI-driven innovations for learning languages, further research is required to fully comprehend how these advancements specifically affect ESP learners, especially those in financial engineering.

Our approach is influenced by social-cultural theory, it holds that instruments are important owing to the way technologies change human behaviour instead of because they possess intangible characteristics [61,62]. According this particular viewpoint, the involvement of techniques in behaviour changes the whole framework and progression of cognitive processes while only enabling measures that would have been possible regardless of devices. Therefore, during 1990s, applied linguistics scholars realized that machine learning had altered almost every aspect of language usage and acquiring knowledge, not just teaching similar material across a different format [63].

By doing rigorous literature review, the various research studies have investigated the effects of AI-driven corpora on EFL learners’ vocabulary learning and lexical bundles. The most of research investigation have found that the comprehension of effective language learning techniques by exploring the possible advantages of integrating AI-driven corpora throughout EFL training. In particular, the findings clearly clarified that how AI-powered tools might improve students’ capacity to recognize and apply pertinent lexical bundles, enabling more efficient engagement in their respective fields of profession. This research study investigates the effects of driven by AI corpora on EFL their inspiration to write, lexical bundles, and vocabulary acquisition. The current study’s findings are expected to provide light on the potential benefits of utilizing AI-driven corpus analysis for EFL instruction. In particular, it is expected that the application of AI-driven technologies will improve learners’ capacity to recognise and apply pertinent lexical bundles, leading to more efficient communication in their line of work. Additionally, the study aims to ascertain how AI-driven corpora impact students’ willingness to write.

Specifically, it is anticipated that the use of AI-driven technologies will enhance learners’ ability to identify and utilize relevant lexical bundles, thereby facilitating more effective communication within their professional domain. The research also seeks to determine how the willingness of students to compose writing is affected by AI-driven corpora. The following research questions were tackled in accordance alongside the research gap that now exists:

1. Does the learning grasp and retention of lexical bundles by students learning EFL become significantly impacted by artificial intelligence-driven corpora?
2. Does the acquisition of grammatical structures by students of EFL become significantly impacted by artificial intelligence-driven corpora?
3. Does Artificial Intelligence Driven Corpus have significant effect on ESP learners’ willingness to write?

By going through the existing research studies along with examining how AI-driven corpora affect EFL learners’ acquisition of vocabulary, lexical bundles, and writing motivation, the current study fills this research gap. By examining the potential benefits of incorporating AI-driven corpora into EFL instruction, the study contributes to our understanding of effective language learning strategies. Specifically, the findings are expected to shed light on how AI-driven technologies can enhance learners’ ability to identify and utilize relevant lexical bundles, facilitating more effective communication within their professional domain.

## 3. Methodology

### 3.1. Design and Sampling

To find out how the intervention affected the lexical groups, grammatical comprehension, and readiness to writing EFL (English just like Foreign Language) students, an iterative form of quasi-experiment had been used. By using that approach, it was possible to compare a similar cohort of respondents before and after treatment, avoiding the need for randomly assigning to groups. The participants were 80 EFL learners enrolled in the basic writing course at School of foreign languages, Baotou Teachers’ College in China. They were divided into two intact classes, with each class comprising 40 students. Depending on the level of enrolment, the individuals received placement in the experimental or controlled group. Each one of them spoke Chinese as their first language. Everyone understood why this investigation was being conducted. Males made up 60% among the respondents, while females made up 40%. From July 1, 2024, until July 15, 2024, the study was carried out. Baotou Teachers’ College has given it their approval (Approval No. STE-BTTC2024001). Every study protocol followed the 1964 Helsinki Declaration, its subsequent revisions, or similar ethical guidelines. When the study began, participants gave permission in writing. The factors that were investigated were measured using a single scale and a pair of tests: the lexical bundles exam, the grammatical knowledge test, and the motivation to write. The following is a detailed explanation of each:

### 3.2. Willingness to write scale

The WTC questionnaire that was comprised of Eight responses designed by MacIntyre et al. [55], adopted to gauge the EFL the preparedness of students for writing. Cronbach’s alpha technique was employed to evaluate the internal reliability (alpha), as well as the score obtained is 0.83, indicating satisfactory result.

### 3.3. Grammar test

A grammar test comprising 30 multiple-choice items was designed to evaluate learners’ comprehension of clauses, infinitives, gerunds, positives, and prepositions. The content validity of the test was determined through evaluation by three subject matter experts. Reliability analysis was conducted using Kuder-Richardson Formula 21 (KR-21), yielding a coefficient of 0.79 for the pretest and 0.81 for the posttest.

### 3.4. Lexical bundles test

A lexical bundles test comprising 30 multiple-choice items was developed to assess learners’ familiarity with common word combinations and phrases. The test aimed to evaluate learners’ proficiency in recognizing and using lexical bundles effectively. Content validity was evaluated by three experts in the field. Reliability analysis was conducted using Kuder-Richardson Formula 21 (KR-21), yielding a coefficient of 0.79 for the pretest and 0.81 for the posttest.

This computer-based exercise involved creating a questionnaire, entering data into a datasheet, and then using SPSS software to analyze the data and estimate the outcomes. The resources (tests, participants, and questionnaire) were created by reviewing earlier research studies that carried out similar investigations. Similarly, this study used MacIntyre et al.‘s [55] eight-question scale to gauge participants’ “willingness to write.” Additionally, 30 multiple-choice questions for the “Grammar” and “Lexical bundles” evaluations were used to gauge participants’ answers.

Randomised processes, likewise throwing a coin, have been employed for assigning individuals to interventions in randomized research. This means that if participants accept to take to participate during research investigation, they will have the same likelihood of being assigned into the immediate treatment session. subsequently is anticipated which observed and unseen prior to treatment factors are, on average, possess comparable values throughout the entire treatment procedures when randomization is used with a substantial number of samples. Consequently, non-randomized study results which also known as quasi-experiments or observation-based studies have frequently relied upon when examining the causal implications of treatments. While some authors consider observational investigations as well as quasi-experiments constitute separate research strategies. One characteristic shared by observational studies and quasi-experiments is that participants cannot be assigned to treatments randomly [64]. A confounding framework occurs when the possible an inconvenience characteristic are dispersed consistently among conditions when the chosen learners have not been randomised to conditions spontaneously. According to a design approach, intact classes therefore have to be viewed differently from occurrences. Random selection is obviously inadequate to provide probability equality during investigations involving just two classes that remain intact because there merely two experimental component measurements. This is one of the main factors that separate quasi-experimental approach and experimental design [65].

### 3.5. Ethical Statement

The study has been approved by Baotou Teachers’ College (Approval No. STE-BTTC2024001). All procedures performed in studies were in accordance with the 1964 Helsinki Declaration and its later amendments or comparable ethical standards.

### 3.6. Procedure

Both the experimental group and controlled group filled out the WTC Scale and the chosen procedures at the beginning of the investigation. Following this initial assessment, the treatment commenced, consisting of 14 sessions designed to enhance writing skills and increase willingness to write. The treatment incorporated various instructional strategies and activities aimed at improving writing proficiency and fostering a positive attitude towards writing. More particularly, two instructional methods were employed to teach grammar: one utilizing traditional textbooks (control group) and the other leveraging artificial intelligence (AI) for corpus generation (experimental group). For the control group, standard grammar lessons and exercises from textbooks were provided. Learners engaged with traditional instructional materials covering clauses, infinitives, gerunds, and appositives. In contrast, the experimental group received innovative instruction through AI tools. Initially, learners were introduced to AI and its role in corpus generation for language learning. Guided practice sessions familiarized them with AI interaction and corpus generation processes. Subsequently, learners engaged in collaborative exploration and independent experimentation with AI-generated corpus data. They worked together to analyze corpus insights and independently refined their approach based on feedback gained from corpus analysis.

Throughout the sessions, reflection periods prompted learners to discuss their experiences and insights gained from using AI for corpus generation. Participants were urged to incorporate corpus-based understanding across a variety of language-acquisition tasks, including writing assignments and grammatical evaluation. Testing and continuing assistance was given each of the group. While the control group continued with traditional textbook instruction, the experimental group received support for continued utilization of AI tools for corpus generation. Regular evaluations and feedback mechanisms assessed learners’ progress and understanding of grammar structures in both groups. Throughout the treatment period, participants engaged in classroom activities, writing exercises, and collaborative tasks tailored to their proficiency level. Appreciating feedback, forming opinions, controlling emotions, and acting are the four interconnected components of the framework [66]. The interactively teaching feedback model (ITF-model) as well as its application to the development and assessment of feedback tactics for distance education settings [67]. Additionally, the examined strategies to ensure sustained student involvement and suggested the double-loop evaluation model for a solution towards an assortment of challenging experiences learners endured. The similar tool applied during the initial phase of trial served to conduct post-treatment evaluations among the two groups towards the completion of 14 sessions.

**Fig1:**
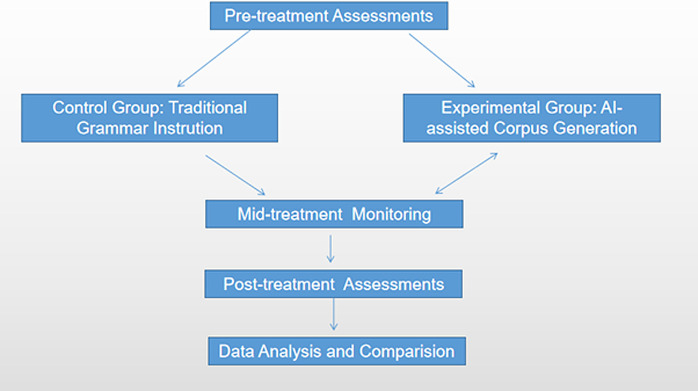
Graphical presentation of the procedure.

The data collected from the pre- and post-treatment assessments were subjected to statistical analysis to determine the effects of the treatment on the participants’ willingness to write and writing proficiency. Specifically, a repeated measures analysis of variance (ANOVA) was conducted to examine within-group differences over time. Additionally, between-group comparisons were made to assess the differential effects of the treatment on the control and experimental groups. Post-hoc tests, such Bonferroni, were employed to further explore significant findings and identify specific areas of improvement. The significance level was set at p < 0.05. The procedure is illustrated in Figure 1

## 4. Results

The following section provides the results, which include inferential as well as descriptive statistics.

The mean results as well as their standard deviations regarding the pretest, posttest, and follow-up test determines for each group are displayed in Table 1. The experimental group exceeded the controlled group (posttest: M = 23.21, SD = 3.23; follow-up: M = 23.21, SD = 3.60) in the lexical bundles domain, demonstrating greater mean results at posttest (M = 17.25, SD = 3.56) and follow-up (M = 17.21, SD = 3.69). In a similar manner the group receiving the experiment outscored the controlled group (posttest: M = 18.50, SD = 2.89; follow-up: M = 18.50, SD = 2.89) across the grammatical knowledge realm, with greater average scores in posttest (M = 24.23, SD = 4.11) and follow-up (M = 24.15, SD = 3.56). Additionally, in terms of communication willingness, the group receiving the experiment outperformed the controlled group (posttest: M = 3.40, SD = 0.79; follow-up: M = 3.26, SD = 0.79) in terms of average results at posttest (M = 4.52, SD = 1.1) and follow-up (M = 4.49, SD = 1.00). These results imply that the experimental group, that was taught using AI-based techniques, performed better and was more open to communication than the counterpart-controlled group, which was taught conventionally. The sum of squares (SS) and mean squares (MS) values can be high relative to the F-values, as given in the findings of Shamsabadi et al. [68]. The outcomes were subjected to Mixed ANOVA in order to assess the hypotheses; Table 2 displays the results.

**Table 1:** Descriptive analysis of the pretest, posttest, and follow-up test results for each group.

	Groups	Pretest		Posttest		Follow up	
M	SD	M	SD	M	SD
Lexical bundles	Control	15.23	3.23	17.25	3.21	17.21	3.60
	Experimental	15.40	3.60	23.23	3.56	23.21	3.69
Grammatical knowledge	Control	17.23	3.70	18.50	2.89	18.50	2.89
	Experimental	17.29	4.11	24.23	4.11	24.15	3.56
Willingness to communicate	Control	3.23	0.80	3.40	0.79	3.26	0.79
	Experimental	3.32	1.1	4.52	1.1	4.49	1.00

**Table 2:** Mixed ANOVA results.

Variables		SS	df	MS	F	p	Eta
Lexical bundles	Time	189.32	2	114.32	42.12	<0.0001	0.64
	Groups	220.16	1	220.16	28.42	<0.0001	0.56
	Time*groups	316.54	2	158.77	59.96	<0.0001	0.46
Grammar test	Time	176.63	2	88.31	20.38	<0.0001	0.68
	Groups	949.58	1	949.58	16.47	<0.0001	0.32
	Time*groups	277.74	2	138.87	32.05	<0.0001	0.48
Willingness to communicate	Time	189.32	2	94.66	35.75	<0.0001	0.42
	Groups	220.16	1	220.16	28.42	<0.0001	0.40
	Time*groups	317.54	2	158.77	59.96	<0.0001	0.35

A sequence of events, the implications of time and group membership on lexical bundles, grammar test scores, and communication willingness were investigated using ANOVA. The results revealed significant main effects of time for lexical bundles, F (2, x) = 42.12, p < 0.0001, η² = 0.64; grammar test scores, F (2, x) = 20.38, p < 0.0001, η² = 0.68; and willingness to communicate, F (2, x) = 35.75, p < 0.0001, η² = 0.42. Additionally, significant main effects of group membership were found for lexical bundles, F (1, 77) = 28.42, p < 0.0001, η² = 0.56; grammar test scores, F (1, 77) = 16.47, p < 0.0001, η² = 0.32; and willingness to communicate, F (1, 77) = 28.42, p < 0.0001, η² = 0.40. Furthermore, significant interaction effects of time and group membership were observed for lexical bundles, F (2, 317.54) = 59.96, p < 0.0001, η² = 0.46; grammar test scores, F (2, 277.74) = 32.05, p < 0.0001, η² = 0.48; and willingness to communicate, F (2, 317.54) = 59.96, p < 0.0001, η² = 0.35. Where “x” represents the correct degrees of freedom for the error term. These findings suggest that both time and group membership have significant effects on language proficiency, as indicated by lexical bundles and grammar test scores, as well as on willingness to communicate. Moreover, the interaction between time and group membership further emphasizes the complexity of these relationships.

## 5. Discussion

The purpose of the study was to find out how AI-driven language corpora affected the lexical bundles, grammatical structures, and writing willingness of EFL learners. Using a repeated measures ANOVA, the study analyzed the effects of time and group membership on these variables. The significant main effect of time on lexical bundles suggests that the intervention, likely involving exposure to AI-driven language corpus, positively influenced learners’ acquisition of lexical bundles over time. This result is consistent with studies by Sun & Wang [60], which showed how beneficial corpus utilization is for vocabulary acquisition. Furthermore, learners who underwent the treatment fared better than students who weren’t, according to the substantial primary impact produced by group. The research results of Kotamjani et al. [69], whose work emphasized the advantages of using online corpus applications in language acquisition, are in support of the results above.

The main outcome of time on grammar test scores, like those of lexical bundles, shows that grammatical competency improved during the treatment timeframe. The beneficial effects of corpus-linguistics research on grammar acquiring, supports this finding [16,19]. Furthermore, the main effect of group underscores the advantage of the intervention group in enhancing grammatical skills, echoing the findings of Crosthwaite et al. [70] on blended learning approaches.

Addressing the third question in this research study, learners’ readiness to communicate increases over time, as indicated by the significantly main impact of time. This could be ascribed to the optimism that comes from better lexical and grammatical competence. This finding is consistent with the research of O’Keeffe [18] and Crosthwaite et al. [4], who highlighted the role of data-driven learning in promoting communicative skills.

The follow-up test scores for the experimental group in the grammatical knowledge section has not appear to show any drop-off or variance, which could be possible because the data was collected through the responses of the participants and their responses may have no significant distinguishing. This can also be the case that asking students to generate grammar structures that they find challenging afterwards addressing their errors might make them feel more anxious and create psychological affective barrier that prevents them from understanding. It was frequently noted that learners who obtained the desired structure using presentation, practice and production (PPP) approach frequently disregard quickly. This is because they fail to acknowledge to concentrate on framework whenever it first appears by their instructor, rendering procedures and execution physical and devoid of rule internalization.

Moreover, the main effect of group membership (suggests that learners exposed to the AI-driven language corpus exhibited higher levels of communication willingness in contrast to the controlled category, highlighting its significance of technologically augmented language learning conditions [71]. Finally, the among the key findings, the significant interaction effects of time and group membership on lexical bundles, grammar test scores, and willingness to communicate underscore the complex interplay between instructional interventions and learner progress. These findings highlight the need for tailored language teaching approaches that integrate AI-driven technologies with traditional pedagogical methods, as advocated by Boulton & Cobb [72].

Future research might set specialised labs where learners might experience videogames in an identical manner, or educators might implement better approaches to instruction involving small class sizes to foster more interpersonal interaction. The majority of their distinctive qualities may nevertheless result in variations in classroom participation even when there has been no apparent distinction in their academic abilities or methods of instruction while they maintained consistency in the classroom material. Upcoming quasi-experimental research ought to verify that all groups have an identical trainer. In addition, because there was neither validated English assessment at large scale, the learner’s post-experiment language proficiency cannot be comparable.

## 6. Limitations

The research’s internal and external reliability remains susceptible to some limitations in methods applied. Initially this research was held in an educational institution within a region of China that was economically prosperous. For a wider generalizability, further investigation is required to extend to underprivileged areas and institutions. Furthermore, only one group across every circumstance may be a confounding variable. In connection with that, there aren’t many people taking into account in the current research. Therefore, attention must be made considering to how broadly applicable such situational results appear. Further research is required to figure out the usefulness of AI-driven corpora and learners’ effectiveness, thus we suggest a larger sample of teachers as well as learners having different backgrounds and levels of competence in English to enable to produce highly useful and reliable data. Furthermore, because classrooms usually had more than 30 students—some even more than 40—teachers were unable to give all learners the same amount of attention.

As a result, instructors spend greater amounts of time upholding control, frequently ignoring underprivileged pupils. Growth of learners is expected to vary depending on their socioeconomic background, gender as well, and cognitive ability. Additionally, the assessment was not created with those learning a second language in mind. The third-grade learners received a test that was designed for kindergarteners who were learning English for their first language with the intent to regulate their learning concern’s challenges, which somewhat skewed their skill levels. There exists a significant demand for research on how flipped educational environments affect the ability of learners for reasoning and language used to communicate.

## 7. Recommendation and suggestions insights

Lessons learnt from this research investigation point to many recommendations for language education. AI-driven language corpora can be incorporated pedagogically by teachers to improve students’ vocabulary, grammar, and communication abilities. This emphasizes how important dynamic and captivating learning environments are. Curriculum designers ought to think about implementing data-driven learning strategies for a range of language proficiency levels. Future studies can determine best-suited approaches to education and examine the long-term impacts regarding these interventions.

Ensuring that all learners take advantage of the latest developments in language teaching requires policies that promote fair access to technologically improved language learning materials. Using AI-driven language corpora can, in general, enable students to become more proficient and self-assured in their language abilities, leading to more successful language teaching methods. In suggestion, improving sustainable development because this relates to learners’ perpetual growth serves as a crucial educational objective. We anticipate that administration’s learning actions to strengthen AI-driven corpora remains in the spirit of broad agenda as well as, requires more scientific research to raise the standard of educational institutions, that could influence execution and improvement inside and outside of China.

## 8. Conclusions and implications

The results of the present investigation demonstrate how driven by AI language corpora have a major influence on EFL students’ willingness to engage in conversation, grammatical structure, and lexical bundle acquisition. The considerable main effects of time on lexical bundles, grammar test scores, and communication willingness demonstrate that the intervention produced significant changes over time. Furthermore, the most significant impact of belonging to groups across such factors showed that learners who underwent the treatment performed better than control cohort. Moreover, the interaction effects of time and group membership further underscore the nuanced relationship between instructional interventions and learner outcomes. According to the findings, the experimental group—which received instruction based on artificial intelligence—performed better and was more receptive to communication than the counterpart-controlled group, which received traditional instruction. These results imply that language skill, as measured by lexical bundles and grammar exam scores, as well as communication desire, are significantly impacted by both time and group participation. The intricacy of these linkages is further highlighted by the interplay between time and group membership.

## Supporting information

S1 Table Some Lexical bundles. (DOCX)

S1 File Data that support the research. (DOCX)
