# Supplementary material for: AI-generated corpus learning and EFL learners’ learning of grammatical structures, lexical bundles, and willingness to write
Source: PLoS One. 2025 Jul 11;20(7):e0321544. doi: 10.1371/journal.pone.0321544 (PMC12250547; doi:10.1371/journal.pone.0321544)
Supplement: S1 Table — (DOCX) [file pone.0321544.s001.docx]

**Lexical bundles:**

**Control Group:**

- **Pretest:**
  - Scores: [16.83, 14.78, 17.32, 20.15, 14.47, 14.47, 20.33, 17.71, 13.71, 16.98, 13.73, 13.73, 16.01, 9.05, 9.66, 13.41, 11.96, 16.25, 12.30, 10.67, 19.96, 14.50, 15.45, 10.63, 13.47, 15.59, 11.51, 16.44, 13.29, 14.29, 13.29, 21.21, 15.19, 11.81, 17.89, 11.29, 15.90, 8.90, 10.94, 15.87]
- **Posttest:**
  - Scores: [17.62, 15.78, 14.86, 14.26, 10.45, 12.90, 13.74, 18.64, 16.34, 9.54, 16.28, 13.99, 13.04, 17.21, 18.56, 18.24, 12.52, 14.23, 16.30, 18.38, 13.68, 14.63, 11.66, 11.37, 17.85, 19.61, 15.00, 18.47, 16.40, 13.15, 16.40, 20.20, 15.11, 20.28, 6.77, 17.88, 15.51, 14.26, 15.53, 8.81]
- **Follow-up:**
  - Scores: [15.10, 17.18, 21.21, 14.02, 12.98, 14.08, 19.19, 17.07, 13.98, 17.74, 16.24, 19.38, 13.36, 14.71, 19.82, 19.69, 16.88, 19.71, 19.32, 19.30, 16.67, 12.99, 15.75, 13.68, 19.52, 19.53, 12.70, 18.91, 10.95, 10.65, 17.28, 16.47, 16.30, 16.65, 12.95, 16.24, 16.46, 12.83, 22.12, 17.11, 11.11, 17.76]

**Experimental Group:**

- **Pretest:**
  - Scores: [19.76, 26.03, 27.35, 20.31, 26.66, 24.70, 26.16, 29.98, 22.36, 20.55, 20.06, 20.33, 22.96, 24.44, 24.22, 26.17, 23.28, 28.40, 22.29, 32.91, 25.46, 20.18, 19.42, 24.95, 22.43, 25.77, 24.91, 22.97, 20.22, 17.84, 21.64, 26.28, 23.99, 18.80, 23.85, 24.60, 20.08, 23.78, 23.44, 19.16]
- **Posttest:**
  - Scores: [24.53, 25.28, 27.21, 27.10, 18.13, 19.75, 25.11, 25.11, 25.11, 37.43, 25.32, 27.40, 26.73, 25.61, 22.05, 26.01, 20.36, 22.34, 21.42, 23.51, 31.75, 16.32, 25.74, 17.26, 21.47, 27.23, 23.45, 19.23, 20.57, 25.72, 20.51, 24.01, 23.38, 20.81, 31.12, 25.55, 15.74, 23.90, 20.77, 26.36]
- **Follow-up:**
  - Scores: [26.15, 19.65, 21.72, 21.70, 25.94, 26.35, 25.94, 19.29, 27.23, 22.32, 25.06, 26.83, 24.48, 18.87, 22.58, 22.99, 23.55, 17.73, 23.11, 25.31, 18.81, 21.71, 25.94, 18.55, 29.08, 28.58, 22.80, 24.30, 24.65, 26.05, 21.74, 20.83, 23.17, 22.01, 24.98, 28.18, 24.00, 22.30, 20.71, 24.16]

Grammatical knowledge

**Control Group:**

- **Pretest:**
  - Scores: [14.30, 16.81, 19.10, 20.43, 12.79, 15.99, 15.47, 14.81, 23.76, 18.73, 12.56, 20.63, 25.08, 21.05, 11.61, 15.44, 21.92, 14.61, 18.87, 20.10, 13.80, 17.01, 5.24, 13.44, 16.30, 12.61, 23.27, 11.94, 15.60, 17.71, 22.56, 11.92, 21.53, 17.27, 13.60, 18.94, 17.97, 15.01, 17.49, 15.80]
- **Posttest:**
  - Scores: [18.83, 20.41, 23.08, 14.92, 24.66, 12.86, 18.06, 20.20, 19.31, 16.70, 17.90, 17.08, 16.80, 20.96, 19.53, 16.50, 21.10, 19.39, 20.85, 20.32, 16.10, 16.88, 20.66, 20.26, 18.44, 18.84, 22.19, 16.79, 20.08, 17.92, 17.87, 21.68, 20.89, 20.85, 22.27, 18.56, 20.47, 17.60, 19.44, 18.12]
- **Follow-up:**
  - Scores: [18.78, 20.22, 16.14, 24.55, 15.59, 14.99, 21.85, 20.79, 20.30, 20.32, 18.46, 15.91, 18.72, 16.54, 18.60, 18.92, 18.32, 18.67, 17.56, 17.52, 18.48, 20.40, 15.92, 17.90, 14.88, 18.89, 16.72, 20.11, 14.33, 15.24, 21.33, 14.60, 21.85, 18.01, 21.40, 18.90, 18.40, 20.22, 22.27, 19.37]

**Experimental Group:**

- **Pretest:**
  - Scores: [15.96, 15.01, 13.76, 16.83, 10.48, 17.69, 17.93, 19.29, 16.30, 20.34, 17.98, 21.43, 18.85, 19.52, 15.25, 14.72, 19.12, 19.22, 17.15, 16.79, 22.06, 16.59, 19.90, 22.18, 22.63, 13.47, 18.52, 21.54, 22.85, 17.16, 19.36, 13.20, 20.73, 22.02, 18.06, 18.90, 23.76, 14.65, 15.69, 15.62]
- **Posttest:**
  - Scores: [21.77, 24.25, 24.42, 22.38, 26.79, 19.84, 23.64, 24.72, 26.34, 27.15, 19.61, 17.92, 29.48, 25.60, 21.15, 30.61, 24.71, 29.08, 24.51, 32.70, 31.44, 23.21, 28.22, 26.88, 29.86, 20.26, 27.05, 28.58, 17.00, 19.37, 15.85, 23.12, 27.18, 30.40, 24.53, 30.92, 18.56, 17.23, 24.00, 25.52]
- **Follow-up:**
  - Scores: [25.52, 24.03, 16.79, 23.83, 19.51, 26.53, 25.46, 20.80, 22.32, 20.38, 23.93, 27.55, 20.64, 25.94, 22.26, 21.33, 23.77, 20.46, 22.18, 19.89, 31.14, 24.28, 21.66, 24.91, 23.75, 23.36, 26.34, 26.85, 22.26, 22.10, 23.17, 15.96, 18.76, 29.02, 30.01, 23.26, 26.20, 25.26, 35.11, 28.14]

Willingness to communicate

**Control Group:**

- **Pretest:**
  - Scores: [3.13, 2.47, 1.94, 3.39, 2.62, 2.09, 2.71, 2.36, 4.58, 3.94, 3.22, 4.41, 3.29, 2.54, 4.45, 3.66, 2.40, 3.08, 2.53, 2.12, 3.97, 4.76, 2.11, 3.68, 2.71, 2.84, 2.76, 2.54, 3.27, 2.57, 3.45, 3.19, 3.04, 2.50, 2.77, 3.83, 3.63, 2.45, 3.31, 3.83]
- **Posttest:**
  - Scores: [2.08, 3.83, 2.88, 3.85, 2.80, 1.97, 2.11, 3.44, 3.61, 2.69, 3.90, 2.09, 3.35, 2.44, 2.89, 3.44, 2.72, 3.10, 4.19, 2.94, 4.06, 2.51, 3.82, 4.54, 1.45, 2.77, 3.86, 3.24, 3.69, 2.92, 3.47, 3.28, 4.32, 3.60, 3.67, 3.07, 3.01, 3.06, 3.71, 3.07]
- **Follow-up:**
  - Scores: [3.49, 4.90, 3.95, 3.00, 4.21, 2.94, 1.65, 2.46, 1.78, 2.98, 3.27, 4.58, 3.52, 3.09, 3.92, 2.91, 4.41, 2.49, 3.49, 2.20, 2.88, 2.83, 3.10, 4.00, 1.48, 2.30, 2.95, 3.64, 2.98, 2.35, 3.10, 2.66, 2.75, 2.23, 2.47, 4.03, 4.08, 1.90, 2.23, 1.90]

**Experimental Group:**

- **Pretest:**
  - Scores: [3.16, 3.25, 2.93, 2.27, 4.10, 4.41, 1.73, 3.90, 1.62, 3.32, 2.53, 3.54, 3.32, 2.48, 4.67, 3.92, 3.22, 4.14, 2.36, 1.58, 2.88, 2.18, 3.36, 3.73, 3.80, 4.50, 2.87, 2.75, 4.61, 3.72, 1.73, 3.26, 3.13, 2.75, 3.33, 3.73, 2.45, 4.37, 3.57, 1.78]
- **Posttest:**
  - Scores: [4.15, 6.36, 4.23, 2.87, 4.25, 4.22, 1.55, 4.46, 4.27, 5.29, 6.55, 5.76, 4.22, 3.30, 7.35, 4.59, 4.54, 4.49, 4.74, 4.36, 3.89, 3.92, 4.48, 3.92, 3.74, 4.64, 4.24, 6.17, 1.60, 5.72, 5.89, 2.24, 4.14, 4.11, 2.97, 3.66, 3.30, 6.45, 5.55, 5.92]
- **Follow-up:**
  - Scores: [5.21, 3.36, 3.97, 4.98, 3.27, 5.20, 4.25, 4.12, 5.20, 4.93, 4.13, 5.65, 3.41, 5.11, 5.08, 4.18, 4.82, 3.24, 5.41, 4.31, 3.97, 5.54, 3.79, 3.08, 2.93, 5.10, 3.21, 6.24, 2.41, 6.19, 4.70, 4.39, 3.95, 4.89, 4.45, 5.59, 4.60, 4.64, 4.13, 4.43]
