# Supplementary material for: AI-generated corpus learning and EFL learners’ learning of grammatical structures, lexical bundles, and willingness to write
Source: PLoS One. 2025 Jul 11;20(7):e0321544. doi: 10.1371/journal.pone.0321544 (PMC12250547; doi:10.1371/journal.pone.0321544)
Supplement: S1 File — (DOCX) [file pone.0321544.s002.docx]

**S1 Table.** Some Lexical bundles

| on the other hand | as a result of |
| --- | --- |
| one of the most | when it comes to |
| is no doubt that | are a lot of |
| there is no doubt | the most important |
| is one of the | there are a lot |
| have to agree that | in the past the |
| i have to agree | a lot of time |
| one i have to | in the past was |
| one of the main | at the same time |
| a lot of people | have a lot of |
| first there is no | will not be able |
| will be able to | in front of the |
| a clear indicator that | there are some differences |
| is a clear indicator | if you want to |
